# Genetically informed therapy for valvular heart disease: what, where, and when to target

**DOI:** 10.1016/j.ebiom.2026.106529

**Published:** 2026-10-20

**Authors:** Yuze Mi, Yanggang Hong

**Affiliations:** a Wenzhou Medical University, Wenzhou, Zhejiang, China; b The Second Affiliated Hospital and Yuying Children's Hospital of Wenzhou Medical University, Wenzhou, Zhejiang, China

Treatment of valvular heart disease (VHD) has advanced substantially through surgical and transcatheter valve repair and replacement. However, effective pharmacological therapies that directly modify valve pathobiology remain lacking for most forms of VHD.^1^ In this issue of *eBioMedicine*, *Moura-Ferreira* et al.^2^ bring genetics and therapeutics together by linking VHD-associated variation to target tractability, existing drugs, and clinical and experimental evidence. Among 431 candidate genes with available tractability information, only 51 were linked to existing compounds, corresponding to 388 pharmacological agents. This gap is as informative as the candidate list itself: the central question is no longer simply which targets are genetically implicated, but which mechanism should be perturbed, in which direction, and at what stage of disease.

Human genetics can reduce uncertainty in target selection, particularly when the causal gene is assigned with high confidence.^3^ Yet genetic support does not specify a complete treatment strategy. A genome-wide association study locus is not itself a drug target, and a drug may perturb a protein in the opposite direction to a protective genetic effect or influence several proteins simultaneously. Each nomination therefore needs evidence linking variant to gene, gene to functional consequence, and intervention to the protective direction of modulation.^4^ Tissue and cellular context matter equally: the relevant mechanism may act in circulating pathways, valvular endothelial cells, interstitial cells, immune cells, or combinations of these compartments.

This need is especially clear across valve phenotypes. *Moura-Ferreira* et al.^2^ found that 44 of 51 drug-linked genes were associated with aortic valve disease or aortic stenosis (AS), whereas evidence for mitral, tricuspid, and rheumatic valve disease was much thinner. This imbalance may reflect the maturity of the underlying data rather than lesser therapeutic potential. A multi-ancestry genome-wide association study of 86,864 AS cases integrated human aortic-valve expression quantitative trait loci with genetic association data and prioritised genes for functional testing; silencing *CMKLR1* or *LTBP4* in human valvular interstitial cells reduced calcification.^5^ In parallel, imaging-derived valve traits combined with an AS genome-wide association study identified 166 loci, while Mendelian randomisation supported potential causal roles for lipoprotein(a) and low-density lipoprotein in aortic valve dysfunction.^6^ Extending genetics, quantitative phenotyping, valve-specific molecular profiling, and functional validation to other valve disorders could broaden therapeutic discovery beyond the aortic valve. VHD should therefore not be treated as a single molecular indication; relevant targets, cell types, and mechanisms are likely to differ across calcific, myxomatous, rheumatic, and regurgitant disease.

Timing may be just as important as target selection. Genetic associations often reflect lifelong susceptibility, whereas clinical trials commonly begin after structural disease is established. In calcific AS, intensive atorvastatin did not slow disease progression, and neither denosumab nor alendronic acid reduced progression of aortic valve calcification.^7^, ^8^ These findings illustrate that a plausible pathway and an available drug do not by themselves define the therapeutic window. An initiating mechanism may become less modifiable once fibrosis, osteogenic reprogramming, extracellular-matrix remodelling, and calcification become self-sustaining. Prevention of disease onset and treatment of established valve disease should therefore be evaluated as distinct therapeutic windows. Lipoprotein(a) provides a useful example: human genetics strongly implicates this pathway in valve calcification and AS,^9^ while the ongoing Lp(a)FRONTIERS CAVS trial (NCT05646381) is testing pelacarsen in patients with elevated lipoprotein(a) and mild-to-moderate calcific AS.

The next task is to turn prioritisation into a staged translational programme. Candidates should move into valve-specific experimental systems under mechanical and inflammatory conditions, followed by prospective studies enriched for the stage at which the mechanism is expected to remain active. Trial design should separate target engagement from valve modification: serial measurements of valve anatomy and function are needed to distinguish a true valve effect from broader cardiovascular benefit. Quantitative imaging, haemodynamic progression, symptoms, exercise capacity, and time to valve intervention can then be layered according to disease stage. The ataciguat programme provides one example of progression from mechanistic rationale and experimental validation to randomised testing with a valve-specific endpoint.^10^ Such designs would make negative trials more informative by revealing whether failure arose from the target, direction of perturbation, treatment window, or insufficient modification of the valve itself.

*Moura-Ferreira* et al.^2^ provide more than a catalogue of repurposing opportunities. The path forward is to match a credible genetic mechanism to the correct direction of perturbation, relevant valve phenotype and cell context, and responsive stage of disease. Human genetics can identify where to look; successful disease modification will depend on knowing what to perturb, where, in which direction, and when.

## Contributors

Yuze Mi and Yanggang Hong wrote, read, and approved the final version.

## Declaration of interests

The authors declare no conflicts of interest.
